# Growth and Adaptation of Newly Graduated Nurses Based on Duchscher’s Stages of Transition Theory and Transition Shock Model: A Longitudinal Quantitative Study

**DOI:** 10.3390/nursrep15120437

**Published:** 2025-12-09

**Authors:** Lynette Cusack, Loren Madsen, Judy Boychuk Duchscher, Wenpeng You

**Affiliations:** 1 Adelaide Nursing School, The University of Adelaide, Adelaide, SA 5005, Australia; 2 Calvary Health Care, 207 Wakefield Street, Adelaide, SA 5000, Australia; loren.madsen@calvarycare.org.au; 3 School of Nursing, Thompson Rivers University, Kamloops, BC V2C 0C8, Canada; 4 School of Nursing and Midwifery, Western Sydney University, Sydney, NSW 2751, Australia

**Keywords:** graduate nurses, professional development, first-year transition, clinical confidence, longitudinal study

## Abstract

**Background:** The transition from student to registered nurse is a vulnerable period characterised by emotional strain, role ambiguity, and transition shock. Although Graduate Nurse Transition Programs (GNTPs) aim to strengthen early practice readiness, few evaluations use longitudinal, theory-informed approaches or validated tools. **Aim:** To examine the professional role development of new graduate nurses (NGNs) across three transition stages within a major Australian health service. **Design and Methods:** A longitudinal quantitative study guided by Duchscher’s Stages of Transition Theory and the Transition Shock Model. A customised 75-item questionnaire—adapted from the Professional Role Transition Risk Assessment Instrument and the Professional and Graduate Capability Framework—was administered at three transition points (March 2020–March 2021). Four domains were assessed: Responsibilities, Role Orientation, Relationships, and Knowledge and Confidence. Descriptive statistics, Principal Component Analysis (PCA), chi-square tests, and multinomial logistic regression identified developmental patterns and predictors of transition stage. **Results:** PCA supported a four-factor structure consistent with the theoretical domains, explaining 62% of variance. Significant stage-based improvements were found in clinical decision-making (RS6, *p* = 0.005), managing pressure (RS11, *p* = 0.003), leadership perception (RO5, *p* = 0.001), and emotional regulation (RL20, *p* < 0.001). Regression analysis identified role confusion (RS7, χ^2^ = 18.112, *p* = 0.001), leadership potential (RL1, χ^2^ = 25.590, *p* < 0.001), workplace support (RL16, χ^2^ = 12.760, *p* = 0.013), and critical thinking confidence (KN13, χ^2^ = 10.858, *p* = 0.028) as strong predictors of transition stage. By Stage 3, most NGNs demonstrated increased autonomy, confidence, and professional integration. A coordinator-to-graduate ratio of 1:12 facilitated personalised mentorship. **Conclusions:** Findings provide robust evidence for theoretically grounded GNTPs. Tailored interventions—such as early mentorship, mid-stage stress support, and late-stage leadership development—can enhance role clarity, confidence, and workforce sustainability.

## 1. Introduction

The transition from student to professional nurse represents a critical juncture that strongly influences long-term outcomes such as job satisfaction, clinical competence, and workforce retention [1,2,3,4,5]. This period is often marked by a rapid escalation in responsibility, emotional strain, and adjustment to complex professional roles [6,7]. New graduate nurses (NGNs) frequently report feeling underprepared for clinical realities, experiencing what Duchscher (2009) terms “transition shock” [8], characterised by cognitive overload, emotional instability, and temporary loss of confidence [9]. These early workplace encounters shape individual career trajectories and affect the stability and sustainability of the nursing workforce [10,11,12].

To address these challenges, healthcare systems have implemented Transition to Practice Programs (TTPPs) and Graduate Nurse Transition Programs (GNTPs) [13,14,15], which provide structured orientation, supervision, professional development, and mentorship opportunities [11,16]. Evidence shows that such programs improve clinical performance [17], enhance confidence and professional identity [18], reduce attrition [19], and improve patient care quality [20]. However, despite their benefits, many GNTPs lack consistent theoretical foundations, and their long-term impact remains under-evaluated through rigorous longitudinal, quantitative methods [11]. Few studies have tracked NGNs across multiple transition stages, and most available evidence is cross-sectional or qualitative, limiting understanding of how role clarity, confidence, and workplace integration unfold over time. There is also limited empirical validation of Duchscher’s Stages of Transition Theory and minimal use of integrated, theoretically aligned measurement tools.

Duchscher’s Stages of Transition Theory and Transition Shock Model are widely recognised frameworks describing NGNs’ progression through “Doing,” “Being,” and “Knowing”—stages characterised by task-focused survival, role internalisation, and emerging autonomy [21,22,23]. Although influential in policy and program design, few empirical studies have examined NGNs’ movement through these stages or the domain-specific factors predicting successful transition. Much existing work is qualitative or cross-sectional, restricting insight into how professional growth evolves over time or how transition frameworks operate in practice [24].

Recent reviews emphasise the need for theory-driven, longitudinal approaches to capture the complexity of NGN development [17]. Edwards et al. (2015) highlight key dimensions requiring monitoring—including role clarity, decision-making, workplace relationships, and emotional regulation—which are particularly relevant in high-pressure acute care settings where early-career stress is heightened [25,26]. Yet most evaluations still rely on single time-point designs, offering snapshots rather than developmental trajectories. Few studies follow graduates longitudinally across defined transition stages, and no known research has synthesised the Professional Role Transition Risk Assessment Instrument with the Professional and Graduate Capability Framework to create a robust, domain-based tool suitable for longitudinal testing.

These gaps highlight the need for longitudinal, theory-driven research using validated measurement tools. To address this, we developed a customised 75-item survey aligned with Duchscher’s theoretical domains—Responsibilities, Role Orientation, Relationships, and Knowledge and Confidence—to capture stage-based transition patterns.

The present study provides a longitudinal evaluation of a GNTP within a major Australian health service across public and private hospitals. The program was intentionally designed around Duchscher’s principles and supported by policy-directed interventions from Graduate Nurse Coordinators, preceptors, and clinical educators. The customised survey was administered across three transition stages (Stage 1: March–July 2020; Stage 2: July–October 2020; Stage 3: October 2020–March 2021), assessing four domains central to professional development.

The study aimed to evaluate how NGNs’ perceptions and capabilities evolve during the first year of practice by (1) tracking changes across the four domains, (2) identifying statistically significant differences between transition stages, and (3) determining predictors of successful transition. In doing so, it offers data-driven validation of a theory-informed transition framework and new insights into NGN development within complex healthcare environments [27].

## 2. Materials and Methods

### 2.1. Design and Framework

This study used a quantitative design embedded within a structured Graduate Nurse Transition Program (GNTP) informed by Duchscher’s Stages of Transition Theory and the Transition Shock Model. A customised 75-item survey—adapted from the Professional Role Transition Risk Assessment Instrument and the Professional and Graduate Capability Framework—was administered at three time points (1, 5, and 11 months) across a 12-month program within a major Australian health service comprising both public and private hospitals. The design enabled assessment of transition-related perceptions and competencies across four domains: Responsibilities, Role Orientation, Relationships, and Knowledge and Confidence. Although the approach drew on Cusack et al. [22], all procedural elements—including survey structure, administration schedule, and analytical techniques—are described in full to ensure clarity and independence.

The theoretical underpinnings of Duchscher’s framework were operationalised through policy directives within the GNTP. Graduate Nurse Coordinators, Nurse Managers, and preceptors supported structured orientation, educational sessions, and systematic evaluation consistent with the model.

#### Longitudinal Structure and Follow-Up

Participation at each survey wave was voluntary. Anonymous unique identifiers enabled linkage of repeated responses without compromising confidentiality. A total of 72 participants completed the Stage 1 survey, 56 completed Stage 2, and 30 completed Stage 3, reflecting expected attrition across the GNTP while allowing both cross-sectional and longitudinal insights into transition patterns.

Given this variation in participation, the study employed a hybrid longitudinal–cross-sectional design. Repeated responses were analysed within-subjects where possible, while differing participation across stages necessitated cross-sectional comparisons for some analyses. This structure allowed examination of both developmental progression among returning participants and broader stage-based differences across the full cohort, providing a comprehensive view of transition experiences within the GNTP.

### 2.2. Survey Instrument and Domains

A customised 75-item survey was developed by synthesising two established frameworks: the Duchscher Professional Role Transition Risk Assessment Instrument [8] and the Professional and Graduate Capability Framework [28,29]. Both frameworks were reviewed item-by-item to identify overlapping constructs related to new graduate nurses’ responsibilities, role clarity, workplace relationships, and application of clinical knowledge. Where conceptual similarities were identified, items were merged or reworded for consistency. Unique items from each framework were retained to ensure comprehensive domain coverage, while redundant, ambiguous, or highly similar items were removed to minimise respondent burden and enhance clarity across domains.

The final survey was organised into four domains central to transition—Responsibilities, Role Orientation, Role Learning, and Knowledge & Confidence. Each item was mapped to one of these domains based on its underlying construct, ensuring alignment with Duchscher’s theoretical principles. This structure provided a domain-based assessment capable of capturing nuanced changes across transition stages.

An emotional wellbeing component was incorporated using items from Cusack et al. [22], reflecting the psychosocial aspects of transition. These items assessed anxiety related to shift changes, difficulty sleeping due to work concerns, workload stress, emotional responses to clinical incidents, and consideration of leaving the workplace or profession. All items utilised a standardised 7-point Likert scale, promoting coherence and supporting consistent statistical treatment across analyses.

To enhance transparency and facilitate replication, the full survey instrument is provided as a Supplementary File S1 (SF Professional Role Transition Tool v1).

### 2.3. Data Collection and Sample

Online surveys were administered via SurveyMonkey™ (2000) at three transition-aligned time points across the 12-month Graduate Nurse Transition Program. New Graduate Registered Nurses (NGRNs) employed in both public and private hospitals within a large national Australian health service were invited to participate using their work email accounts. Surveys were distributed at one month (Stage 1), five months (Stage 2), and eleven months (Stage 3) into the program. Participation was voluntary at each wave, and respondents could complete one or multiple surveys depending on availability. The final sample comprised 158 graduate nurses who completed at least one survey, forming the basis for both cross-sectional and hybrid longitudinal analyses.

### 2.4. Statistical Analysis

Descriptive statistics were first generated to summarise item-level patterns across the three transition stages, reporting means and standard deviations. These analyses provided an overview of how graduate nurses’ perceptions of role clarity, clinical confidence, and workplace integration changed across stages.

Principal Component Analysis (PCA) was then conducted to examine the underlying factor structure of the survey and validate its four conceptual domains: Responsibilities (RS), Role Orientation (RO), Role Learning (RL), and Knowledge and Confidence (KN). PCA was performed separately for each transition stage and for the combined dataset to assess consistency and progression over time. Sampling adequacy was confirmed using the Kaiser–Meyer–Olkin (KMO) statistic, and Bartlett’s Test of Sphericity verified the suitability of the correlation matrix. Components with eigenvalues > 1.0 were retained (Kaiser’s criterion) and varimax rotation was applied to enhance interpretability. Items with factor loadings ≥ 0.40 were assigned to their respective factors, and where cross-loadings occurred (≥0.40 on multiple factors), items were allocated based on theoretical alignment. Items with low or unclear loadings (<0.40) were excluded. This process strengthened the construct validity of the instrument and identified meaningful thematic changes across stages.

For inferential analysis, the original 7-point Likert responses were recoded into three ordinal categories to meet chi-square and logistic regression assumptions: “Disagree” (1–3), “Neutral” (4), and “Agree” (5–7). Chi-square (χ^2^) tests were used to determine whether item response distributions differed significantly across the three transition stages, identifying shifts in development, confidence, and workplace integration.

Multinomial logistic regression was then used to identify predictors of transition stage, with Stage 3 set as the reference category. Each item was analysed individually to assess its association with being in Stage 1 or Stage 2 relative to Stage 3. Odds ratios and 95% confidence intervals were reported, and all models were adjusted for potential confounders. All item-level results were presented to provide a comprehensive overview across domains.

All analyses were conducted using IBM SPSS Statistics (Version 30), with statistical significance set at *p* < 0.05.

## 3. Results

The findings reveal clear stage-based differences in new graduate nurses’ experiences across the three transition points. Consistent patterns emerged across the four domains—Responsibilities, Role Orientation, Relationships, and Knowledge/Confidence—showing progressive development at the group level. Graduates in later stages reported higher levels of clinical confidence, stronger leadership perception, improved workplace integration, and enhanced critical thinking. These results reflect cohort-level shifts across transition stages rather than uniform individual-level change.

**Figure 1:**
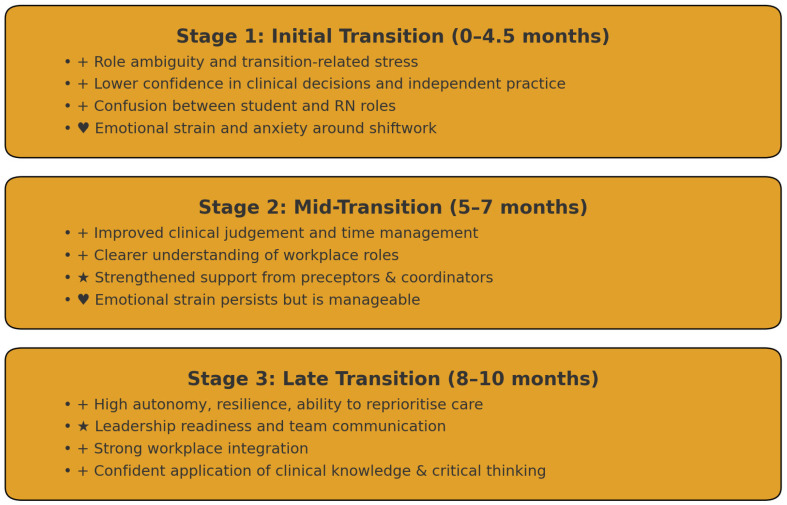
Overview of Role, Skill, and Emotional Development Across Transition Stages. Note: The symbols are used as visual markers to reflect key elements of graduate nurse transition. “+” denotes incre-mental growth in responsibilities, skills, and workplace adaptation; “★” represents emerging competence, confidence, and early leadership potential; and “♥” signifies emotional wellbeing, relational support, and the development of pro-fessional identity during the transition process.

Figure 1 illustrates the overall trajectory. Stage 1 (0–4.5 months) was characterised by role ambiguity, lower confidence in independent practice, and ongoing adjustment to workplace expectations, alongside emotional strain linked to shiftwork and adapting to responsibility. By Stage 2 (5–7 months), graduates demonstrated clearer clinical judgement, improved organisation and prioritisation skills, and stronger relational support from preceptors, coordinators, and peers. Emotional demands remained present but showed signs of stabilisation. Stage 3 (8–10 months) reflected consolidation: participants displayed greater autonomy, resilience, leadership readiness, and stronger alignment with professional identity, alongside increased confidence in applying clinical knowledge.

Across all domains, the data indicate a transition from early uncertainty to more stable professional functioning as graduates progress through the program. These stage-based patterns align with the conceptual progression described in Duchscher’s Stages of Transition Theory. Detailed results, including descriptive statistics, factor structures, chi-square comparisons, and regression findings, are presented in Table 1, Table 2, Table 3 and Table 4.

Table 1 summarises key indicators of transition across the three stages. Overall, the results demonstrate a clear, stage-based progression in clinical confidence, workplace functioning, and professional integration. Graduates reported gradual increases in clinical communication skills, independence in practice, workload management, and responsiveness to changing patient needs, reflecting a steady consolidation of core competencies from Stage 1 to Stage 3.

Interpersonal and team-related indicators also strengthened over time. Perceptions of being respected and accepted within the workplace improved across stages, suggesting enhanced social integration and growing professional identity. Comfort in approaching preceptors and senior staff remained consistently high, indicating strong supervisory support throughout the program.

Emotional stability showed a similar positive pattern, with participants reporting greater calmness under pressure and consistently low intentions to leave the profession. Knowledge-related variables—including confidence in critical thinking—also increased, supported by stable perceptions of organisational backing for ongoing development.

Collectively, these trends illustrate a coherent developmental trajectory: graduates become progressively more confident, better integrated into their teams, and more assured in their professional roles as they advance through the transition stages. Full item-level means and standard deviations for all 75 survey variables are provided in Supplementary Table S1.

Table 2 presents the factor structure of the customised survey, organised around four domains central to new graduate nurses’ transition: Responsibilities, Role Orientation, Role Learning, and Knowledge & Confidence. Each domain represents a distinct aspect of early professional development and illustrates how graduates progress from initial uncertainty toward increasing confidence and workplace integration, with stronger ratings typically observed in later stages.

The Responsibilities domain captures understanding of clinical duties, confidence in everyday practice, workload adaptation, time management, and early autonomy. Together, these factors reflect the shift from role ambiguity in early transition to more consistent and independent clinical functioning.

The Role Orientation domain reflects graduates’ developing clarity around expectations, accountability, and emerging leadership. Items highlight how respect, socialisation, and workplace culture influence professional adjustment and how graduates increasingly understand their role in multidisciplinary teams.

The Role Learning domain includes teamwork behaviours, safe practice processes, supervisory engagement, and emotional adjustment. These factors show how graduates move from reliance on senior staff toward stronger professional identity, improved coping strategies, and increased comfort in the clinical environment.

The Knowledge & Confidence domain assesses preparedness for clinical work, clinical reasoning, understanding of transition processes, and the alignment between expectations and practice. These elements illustrate the transition from reliance on formal education toward confident application of knowledge in real-world settings.

To enhance clarity, Table 2 is organised with sequential domain headings and factor subheadings, allowing readers to follow how items cluster conceptually within each domain. Full item-level factor loadings and associated statistics are provided in Supplementary Table S2.

Table 3 illustrates clear stage-based differences in graduates’ development across the four transition domains. Significant improvements were evident in clinical communication, emotional regulation, leadership readiness, workplace respect, and critical thinking. The most substantial gains appeared in the Responsibilities and Role Learning domains, with additional but selective progress in Role Orientation and Knowledge & Confidence. Collectively, the results reflect a shift from early-stage uncertainty and role confusion toward later-stage professional identity, autonomy, and emotional resilience.

**Responsibilities (RS):** Graduates showed marked progression in core clinical competence. The strongest improvement was reduced role confusion (RS7; χ^2^(4) = 24.477, *p* < 0.001). Growth was also evident in communication and clinical judgement (RS3, RS6, RS9), along with better organisation and stress regulation (RS10, RS11). These patterns reflect a transition from task-focused coping to increased mastery and confidence.

**Role Orientation (RO):** Three indicators showed meaningful development: increased engagement in leadership behaviours (RO5), enhanced perceptions of peer respect (RO6), and improved clinical issue identification (RO12). While other items remained stable, these gains demonstrate strengthening leadership potential and workplace integration.

**Role Learning (RL):** Several elements progressed significantly. Perceived leadership potential rose (RL1), and comfort engaging with senior staff improved (RL7). Increased involvement in clinical incidents (RL18) likely reflects expanded responsibility over time. Emotional indicators (RL20, RL21) also shifted, suggesting that as responsibility grows, some stress persists—reinforcing the need for sustained wellbeing support during transition.

**Knowledge & Confidence (KN):** This domain was generally stable, but critical thinking confidence (KN5) significantly improved (χ^2^ = 11.303, *p* = 0.023). Items approaching significance indicate gradual refinement of applied judgement as clinical exposure increases.

A full list of nonsignificant items is provided in Supplementary Table S3.

Table 4 summarises the multinomial logistic regression findings identifying item-level predictors of transition stage across the four domains: Responsibilities (RS), Role Orientation (RO), Relationships (RL), and Knowledge and Confidence (KN). Transition was categorised into Stage 1, Stage 2, and Stage 3.

In the Responsibilities domain, confusion between the student and registered nurse role (RS7, χ^2^ = 18.112, *p* = 0.001) strongly predicted earlier transition stages, indicating that role clarity strengthens as graduates progress. Confidence in remaining calm under pressure (RS11, χ^2^ = 11.177, *p* = 0.025) was associated with later stages, reflecting the development of emotional regulation. Time management (RS10, *p* = 0.089) and workplace social participation (RS14, *p* = 0.075) showed emerging but nonsignificant trends toward supporting transition progression.

Within the Role Orientation domain, the ability to identify core clinical issues (RO12, χ^2^ = 27.538, *p* < 0.001) was the strongest domain-level predictor, underscoring the importance of developing clinical reasoning. Graduates who balanced work and personal life (RO14, χ^2^ = 10.705, *p* = 0.030), accepted constructive feedback (RO15, χ^2^ = 10.988, *p* = 0.027), or questioned their decision to join the profession (RO17, χ^2^ = 9.545, *p* = 0.049) also demonstrated significant stage variation, reflecting evolving professional identity and self-assessment.

The Relationships domain contained multiple strong predictors of transition progression. Being viewed as a potential leader (RL1, χ^2^ = 25.590, *p* < 0.001) and feeling comfortable approaching coworkers (RL12, χ^2^ = 26.223, *p* < 0.001) were both strongly associated with later stages. Indicators of workplace support (RL16, χ^2^ = 12.760, *p* = 0.013), involvement in clinical incidents (RL18, χ^2^ = 36.640, *p* < 0.001), pre-shift anxiety (RL19, χ^2^ = 10.494, *p* = 0.033), and shift-related sleep difficulty (RL20, χ^2^ = 25.483, *p* < 0.001) also differentiated transition stages. Together, these findings emphasise the combined influence of relational confidence, psychological strain, and increasing responsibility.

In the Knowledge and Confidence domain, feeling adequately prepared by formal education (KN1, χ^2^ = 10.190, *p* = 0.037) and holding realistic expectations of one’s abilities (KN13, χ^2^ = 10.858, *p* = 0.028) were significantly associated with progression to later stages. These results suggest that both perceived readiness and accurate self-evaluation contribute meaningfully to transition advancement.

Overall, Table 4 illustrates that graduate nurse transition is shaped by an interconnected set of cognitive, emotional, relational, and competence-based factors. Enhancing transition support therefore requires multi-domain strategies that foster role clarity, emotional resilience, workplace relationships, and confidence in clinical decision-making.

Only items with statistically significant associations (*p* < 0.05) are presented. Full results for all items are provided in Supplementary Table S4.

## 4. Discussion

This study offers longitudinal, empirical support for Duchscher’s Stages of Transition Theory and the Transition Shock Model [8,21], demonstrating the multidimensional progression that new graduate nurses (NGNs) experience during their first year of practice. The stage-based patterns identified in this study reflect a shift from initial emotional strain, role ambiguity, and cognitive overload toward increasing confidence, autonomy, and professional integration. These findings align with prior research underscoring the vulnerability of early-career nurses and the crucial role of structured transition support in shaping successful professional development, job satisfaction, and retention [11,13,30].

Because participation varied across survey waves, the findings reflect group-level stage patterns rather than uniform within-subject trajectories. Although anonymous identifiers enabled linking repeated responses, the design represents a hybrid longitudinal–cross-sectional structure. As such, references to “growth,” “improvement,” or “development” describe differences between respondents at each stage rather than guaranteed individual change. This distinction is especially relevant when interpreting emotional, cognitive, and professional indicators that may evolve differently across individuals.

Overall, the stage-based differences observed in this study are consistent with the conceptual foundations of Duchscher’s theory. Early stages were characterised by heightened emotional distress, role confusion, and reduced confidence, whereas later stages reflected more consolidated professional identity, stronger relational integration, and greater confidence in clinical decision-making. The alignment between theoretical expectations and empirical patterns reinforces the value of using a domain-aligned, theory-driven measurement instrument to evaluate transition trajectories.

In Stage 1, participants exhibited characteristics typical of transition shock, including low confidence (RS3), role confusion (RS7), and difficulty coping with workplace stressors (RS11). These indicators were statistically significant across stages (RS7: χ^2^ = 18.112, *p* = 0.001; RS11: χ^2^ = 11.177, *p* = 0.025), consistent with Duchscher’s (2008) description of the emotional dissonance and role ambiguity that characterise the early phase of practice [21]. Labrague and McEnroe-Petitte (2018) similarly identified that NGNs commonly experience anxiety and low self-efficacy due to the gap between academic preparation and real-world clinical demands [7]. PCA loadings further confirmed this: RS2 and RS7 loaded on factors related to stress and misalignment across all three phases, underscoring the persistent impact of educational-practice incongruence [17].

Mentorship emerged as a key enabler of progression [30,31]. The 1:12 GNTP coordinator-to-graduate ratio allowed for regular, individualised support, which participants consistently identified as a facilitator of emotional regulation, relational confidence, and workplace integration [22]. Logistic regression revealed that RL10 (support from GNTP coordinator), RL11 (L&D coordinator), and RL12 (peer approachability) significantly predicted progression to later stages (e.g., RL12: χ^2^ = 26.223, *p* < 0.001). These results align with previous studies which found that quality mentorship and consistent access to experienced staff enhance graduate nurse adjustment, confidence, and job satisfaction [32,33,34]. This also emphasised that support structures must be flexible and responsive to individual needs, which was a key feature of the program examined in this study [33,35].

Stage 2 findings showed marked improvements in clinical communication (RS3), self-assessed knowledge (KN5), and the ability to prioritise and analyse information (RO12). These outcomes suggest a shift from coping to consolidation, consistent with findings by Gardiner and Sheen (2016) [36], who reported that mid-transition periods are crucial for reinforcing skills and promoting confidence through team integration. RO12, in particular, emerged as a strong predictor of transition stage (χ^2^ = 27.538, *p* < 0.001), reflecting the increasing importance of critical thinking in professional maturity [37].

Stage 3 reflected a transition into autonomy and professional identity. Participants scored highly on RS13 (care plan adjustment), RO5 (leadership roles), and KN2 (clinical decision confidence), and these items showed high PCA loadings in Phase 3 (e.g., RS13: 0.894; RO5: 0.872). These findings align with Ankers et al. (2018) and Charette et al. (2023), who reported increased decision-making autonomy and leadership emergence as key features of late-stage transition [30,38]. Participants also showed improvements in emotional resilience (RS12) and adaptability (KN4), indicating a move from dependence to competence, as outlined in Duchscher’s “Knowing” stage [39,40].

Despite these advancements, emotional stressors remained evident. RL19 (worry before shifts) and RL20 (sleep disturbances) were significantly associated with earlier stages (RL20: χ^2^ = 25.483, *p* < 0.001), highlighting that psychological burdens can persist even as clinical skills improve. This is supported by Epsteins et al. (2020), who noted that sleep disruption and emotional fatigue often continue throughout the first year, regardless of competence gains [41].

Multinomial logistic regression identified a set of robust predictors across domains. Items such as RS7 (role confusion), RO17 (doubt about career choice), RL1 (perceived leadership potential), and KN1 (perception of academic preparation) significantly predicted stage placement. For instance, RO17 (χ^2^ = 9.545, *p* = 0.049) was associated with questioning one’s career decision, particularly in Stage 1, a pattern also identified by Hunter and Cook (2018), who advocated for incorporating clinical realism into undergraduate education to help manage expectations [42].

Institutional support and interpersonal relationships were also significant. Items reflecting comfort in approaching senior staff (RL7), perceived respect (RO6), and availability of mentorship (RL10–12) were consistently linked with higher transition stages. These results echo Whitehead et al. (2016) and Kaihlanen et al. (2020), who emphasised that feeling respected and supported within teams directly contributes to professional integration [17,43].

Overall, this study contributes empirical evidence to the growing body of literature supporting structured, theory-informed transition programs [44]. The progression observed, from initial anxiety and uncertainty to emerging autonomy and leadership, mirrors findings reported across diverse healthcare settings and highlights the interconnected roles of individual development and institutional support [17,40,45,46].

These findings emphasise the importance of structured, stage-specific support systems that address early emotional stress, foster clinical skill consolidation during the mid-transition phase, and promote leadership development in the later stages to support a successful transition to professional practice [47,48].

The generalisability of these findings is limited by the study’s context within a single Australian health service. Organisational culture, support structures, and staffing models may differ across institutions and regions, potentially affecting transition experiences. Therefore, while the observed developmental trajectories align with established theory and comparable international studies, caution should be exercised in applying these results to graduate nurse cohorts in different healthcare settings or countries.

Because of the hybrid longitudinal–cross-sectional structure, the term “transition trajectory” refers to differences observed across groups at each stage rather than continuous within-subject change for all participants. The use of anonymous identifiers allowed some repeated-response linkage; however, varying participation means these patterns represent aggregated trends rather than definitive developmental progression for every graduate. This distinction is important for interpretation and has been addressed throughout the manuscript by emphasising stage-based differences.

This study should be interpreted in light of several limitations. Although the longitudinal design provides valuable insights into graduates’ progression over time, all data were self-reported and may be influenced by recall bias or social desirability. The sample was drawn from a single Australian health service, which may limit generalisability to other regions or international settings. Attrition across survey waves may also have affected representativeness in later stages, although a substantial proportion of participants contributed data at more than one time point. In addition, while robust statistical techniques such as PCA and multinomial logistic regression were employed, some predictors of transition success may not have been captured due to the item-level modelling approach. Finally, although the survey instrument was adapted from validated frameworks, its psychometric properties may evolve over time and should be re-evaluated in future cohorts.

## 5. Conclusions

This study identified clear stage-based differences in the transition experiences of graduate nurses and midwives, demonstrating how responsibilities, role orientation, workplace relationships, and professional confidence vary across early, mid, and later transition points. Key predictors of transition stage included reduced role confusion, leadership potential, perceived workplace support, and realistic self-assessment of clinical abilities. These findings address the study’s objectives by clarifying which factors align with more advanced stages of transition and how they contribute to professional development.

The results reinforce the value of structured Graduate Nurse Transition Programs grounded in theory and organisational policy. Interventions tailored to each stage—such as early mentorship, mid-stage stress-management strategies, and late-stage leadership opportunities—can strengthen role clarity, confidence, and workplace integration. Sustained organisational investment in GNTPs is therefore essential for supporting workforce capability, enhancing retention, and promoting high-quality, safe patient care.

## Figures and Tables

**Table 1:** Summary of Key Transition Variables Across Three Stages of Graduate Nurse/Midwife Experience.

Key Transition Variable	Stage 1 Mean (SD)	Stage 2 Mean (SD)	Stage 3 Mean (SD)	Trend
Confidence in clinical communication (Calling medical officer)	4.82 (0.97)	5.00 (0.85)	5.20 (0.71)	↑ Improving
Confidence in independent practice	4.59 (0.77)	4.73 (0.65)	5.07 (0.64)	↑ Improving
Ability to manage time and organise workload	4.63 (0.70)	4.82 (0.77)	5.30 (0.60)	↑ Improving
Calmness under pressure	4.47 (0.77)	4.57 (0.63)	5.10 (0.71)	↑ Improving
Understanding of role expectations	4.89 (0.68)	4.98 (0.66)	5.14 (0.64)	↑ Stable/Improving
Feeling respected by colleagues	4.61 (0.87)	4.87 (0.70)	5.17 (0.60)	↑ Improving
Feeling accepted in the workplace	4.81 (0.73)	4.98 (0.63)	5.17 (0.54)	↑ Improving
Comfort approaching preceptors	5.20 (0.88)	5.33 (0.66)	5.52 (0.65)	↑ Strong & Stable
Confidence performing required skills	4.55 (0.79)	4.80 (0.74)	4.97 (0.73)	↑ Improving
Confidence responding to clinical changes	4.39 (0.77)	4.63 (0.65)	4.90 (0.62)	↑ Improving
Workplace support for transition	4.94 (0.74)	5.00 (0.80)	5.17 (0.93)	↑ Stable/Improving
Considering leaving the profession	1.99 (1.21)	2.11 (1.19)	1.90 (0.90)	→ Low & Stable

**Table 2:** Factor Structure and Item Groupings Across the Four Transition Domains.

Domain	Factor	Factor Description	Item Codes (Representative Items)
Responsibilities	R1	Understanding clinical responsibilities	RS1, RS2, RS3
	R2	Confidence in day-to-day practice	RS4, RS5, RS6
	R3	Adapting to workload and patient needs	RS7, RS8, RS9
	R4	Time management and responding to challenges	RS10, RS11, RS12, RS13
	R5	Workplace engagement and work–life boundaries	RS14, RS15, RS16
Role Orientation	O1	Clarity of role expectations	RO1, RO2, RO3
	O2	Accountability and early leadership skills	RO4, RO5
	O3	Respect, socialisation, and workplace belonging	RO6–RO10
	O4	Working with senior staff and decision-making	RO11–RO14
	O5	Adjustment to professional role expectations	RO15–RO17
Role Learning	L1	Workplace acceptance and teamwork	RL1–RL4
	L2	Safety, reporting, and escalation processes	RL5–RL7
	L3	Support from preceptors and coordinators	RL8–RL12
	L4	Professional identity, wellbeing, and self-care	RL13–RL17
	L5	Emotional responses during transition	RL18–RL23
Knowledge & Confidence	K1	Preparedness for clinical practice	KN1–KN3
	K2	Clinical reasoning and judgement	KN4–KN6
	K3	Expectations of clinical practice	KN7–KN10
	K4	Workplace expectations and self-assessment	KN11–KN14
	K5	Understanding of transition theory and shock	KN15–KN16

**Table 3:** Significant Chi-Square Associations Between Transition Items and Training Stage.

Item Code	Item Description	Chi-Square Value	df	*p*-Value
RS3	I am confident calling a medical officer about my patient	9.949	4	0.041
RS6	I am confident with the clinical decisions that I make	14.996	4	0.005
RS7	I sometimes confuse my previous role as a student with my current role	24.477	4	<0.001
RS9	I feel confident caring for patients’ families	9.532	4	0.049
RS10	I am able to organise my work and manage my time effectively	12.401	4	0.015
RS11	I am able to remain calm under pressure or when things go wrong	16.205	4	0.003
RO5	I take a leadership role in my workplace	17.731	4	0.001
RO6	I feel respected by the Nurses/Midwives I work with	13.307	4	0.010
RO12	I can identify the core issue in a clinical situation	17.435	4	0.002
RL1	I am seen as a potential leader in my workplace	26.462	4	<0.001
RL7	I feel comfortable approaching the Clinical Nurse Manager	9.883	4	0.042
RL18	I have been involved in a clinical incident	21.120	4	<0.001
RL20	I find it difficult to sleep between shift changes due to anxiety	12.376	4	0.015
RL21	I have taken Personal Leave due to work-related concerns	9.606	4	0.048
KN5	I feel confident in my ability to think critically	11.303	4	0.023

**Table 4:** Multinomial Logistic Regression Results: Predictors of Transition Stage, Grouped by Domain.

Domain	Item Code	Item Description	Chi-Square	df	*p*-Value
Responsibilities (RS)	RS7	I confuse student and current role	18.112	4	0.001
	RS11	I remain calm under pressure	11.177	4	0.025
Roles (RO)	RO12	I identify the core issue in clinical situations	27.538	4	<0.001
	RO14	I am able to balance personal and work life	10.705	4	0.030
	RO15	I receive constructive feedback without blame	10.988	4	0.027
	RO17	I have questioned my decision to become a Nurse/Midwife	9.545	4	0.049
Role Learning (RL)	RL1	I am seen as a potential leader	25.590	4	<0.001
	RL12	I feel comfortable approaching coworkers	26.223	2	<0.001
	RL16	My workplace supports my transition	12.760	4	0.013
	RL18	I have been involved in a clinical incident	36.640	4	<0.001
	RL19	I worry about responsibilities before shifts	10.494	4	0.033
	RL20	I find it difficult to sleep between shifts	25.483	4	<0.001
Knowledge & Confidence (KN)	KN1	My formal education prepared me well	10.190	4	0.037
	KN13	I have realistic expectations of my abilities	10.858	4	0.028

## Data Availability

The data supporting the findings of this study are available from the corresponding author upon reasonable request, subject to approval by the relevant Human Research Ethics Committee and in accordance with institutional data sharing policies.

## Supplementary Materials

The following supporting information can be downloaded at: https://www.mdpi.com/article/10.3390/nursrep15120437/s1, File S1: Professional Role Transition Tool v1; Table S1: Descriptive Statistics of Key Transition Variables Across Three Stages of Graduate Nurse/Midwife Experience; Table S2: Principal Component Loadings (≥0.40) and Factor Descriptions Across Three Stages; Table S3: Chi-Square Test Results for Survey Items Across Phases: Stage 1, Stage 2, and Stage 3. Table S4: Multinomial Logistic Regression Results: Predictors of Graduate Nurse and Midwife Transition Stage.

## Public Involvement Statement

There was no public involvement in any aspect of this research. Only deidentified data from graduate nursing students were included in the study.

## Guidelines and Standards Statement

This study was designed, conducted, and reported in accordance with established guidelines for quantitative research in the health sciences. The reporting adheres to the STROBE (Strengthening the Reporting of Observational Studies in Epidemiology) checklist for cross-sectional and longitudinal observational studies. All statistical analyses were performed following best practices for psychometric evaluation and multivariable modelling.

## Abbreviations

PCA	Principal Component Analysis
TTPPs	Transition to Practice Programs
GNTPs	Graduate Nurse Transition Programs
RS	Responsibilities
RO	Roles
RL	Relationships
KN	Knowledge
