# Supplementary material for: Growth and Adaptation of Newly Graduated Nurses Based on Duchscher’s Stages of Transition Theory and Transition Shock Model: A Longitudinal Quantitative Study
Source: Nurs Rep. 2025 Dec 9;15(12):437. doi: 10.3390/nursrep15120437 (PMC12736343; doi:10.3390/nursrep15120437)
Supplement: Supplementary file 1 [file nursrep-15-00437-s001.zip › SF File S1 Professional Role Transition Tool v1.pdf]

# Professional Role Transition Tool

This tool was developed by adapting the ***Professional Role Transition Risk Assessment Instrument***© (Dr. Judy Duchscher) and the ***Professional and Graduate Capability Framework***© (Emeritus Professor Geoff Scott).

You will be asked to complete this evaluation survey three times during the next 12 months.

If you suffer any distress as a result of this research project, you should contact the study team as soon as possible and you will be assisted with arranging appropriate support. You are also encouraged to use the Employee Assistance Program (EAP) which is offered free of charge by your local Hospital site. You can source the appropriate contact details of your local Employee Assistance Program through this Calvary Connect link.

Or alternatively utilise this telephone support helpline through the Nurse Midwife Support, which provides free and confidential support 24/7 to all nurses and midwives Australia-wide - 1800 667 877.

| Item Descriptor | Item | Strongly Disagree | Somewhat disagree | Disagree | Agree | Somewhat agree | Strongly Agree | Not Applicable |
|---|---|---|---|---|---|---|---|---|
| | **Responsibilities (RS) 16 Items** | | | | | | | |
| RS1/JS | I understand my responsibilities as a practicing Nurse/Midwife | | | | | | | |
| RS2 | My understanding of my responsibilities as a practicing Nurse/Midwife does differ from what the workplace expects | | | | | | | |
| RS3/CO | I am confident calling a medical officer about my patient | | | | | | | |
| RS4/CO | I am confident speaking to the Team leader about my patient | | | | | | | |
| RS5/CO | I am confident working with Allied Health (Physio, OT, Social Worker) | | | | | | | |
| RS6/CO/DM | I am confident with the clinical decisions that I make | | | | | | | |
| RS7 | I sometimes confuse my previous role as a student with my current role as a practicing Nurse/Midwife | | | | | | | |

| | | | | | | | | |
|---|---|---|---|---|---|---|---|---|
| RS8/CO/CJ/DM | I am confident practicing independently as a Nurse/Midwife | | | | | | | |
| RS9/CO | I feel confident caring for patients families | | | | | | | |
| RS10/CO | I am able to organise my work and manage my time effectively | | | | | | | |
| RS11/CO | I am able to remain calm under pressure or when things go wrong | | | | | | | |
| RS12/CO | I am willing to persevere when things are not working out as anticipated | | | | | | | |
| RS13/CO | I am able to readjust my plan of care in light of what happens when it is implemented | | | | | | | |
| | **Responsibilities (RS) Emotional Assessment 3 items** | | | | | | | |
| RS14/JS | I participate in the social culture of my work place (i.e. socialise with your coworkers) | | | | | | | |
| RS15/JS | I think about work on my days off | | | | | | | |
| RS16/ITS | There are times I have considered leaving my current workplace | | | | | | | |
| | **Roles (RO) 17 Items** | | | | | | | |
| RO1 | I understand what others expect of me (i.e., Clinical Nurse Manager, Team Leader, Coworkers, Assistant in Nursing) | | | | | | | |
| RO2/JS | I understand the difference between my role and an Assistant in Nursing (AIN) | | | | | | | |
| RO3/JS | I understand the difference between my role and that of my senior nurse coworkers (Clinical Nurse Manager, Clinical Nurse, Team Leader) | | | | | | | |
| RO4/CT | I understand the difference in **accountability** between a | | | | | | | |

| | | | | | | | | |
|---|---|---|---|---|---|---|---|---|
| | Registered Nurse/Midwife and an Enrolled Nurse | | | | | | | |
| RO5 | I take a leadership role in my workplace | | | | | | | |
| RO6/JS | I feel respected by the Nurses/Midwives that I work with | | | | | | | |
| RO7/JS | I feel respected by the Assistant in Nurses that I work with | | | | | | | |
| RO8/JS | I feel respected by the non-clinical staff that I work with | | | | | | | |
| RO9/JS | I feel respected by the Medical Officers that I work with | | | | | | | |
| RO10/JS | My role as a Nurse/Midwife is what I thought it would be like | | | | | | | |
| RO11 | I am able to work with senior staff without feeling intimidated | | | | | | | |
| RO12 | I am able to identify from a mass of detail /information the core issue in any situation | | | | | | | |
| | **Roles (RO) Emotional Assessment 5 items** | | | | | | | |
| RO13 | I have a sense of humour and am able to keep work in perspective | | | | | | | |
| RO14/JS | I am able to balance my personal life with my work life | | | | | | | |
| RO15 | I am able to receive constructive feedback from coworkers without feeling any personal blame | | | | | | | |
| RO16/JS | Moving from student to Nurse/Midwife was more difficult than I expected | | | | | | | |
| RO17/ITS | There are times when I have questioned my decision to become a Nurse/Midwife | | | | | | | |
| | **Relationships (RL) 24 Items** | | | | | | | |
| RL1 | I am seen as a potential leader in my workplace | | | | | | | |
| RL2/JS | I feel accepted by the Nurses/Midwives that I work with | | | | | | | |
| RL3/JS | I feel accepted by the Assistants in Nursing that I work with | | | | | | | |

| | | | | | | | |
|---|---|---|---|---|---|---|---|
| RL4/JS | I feel accepted by the Medical Officers that I work with | | | | | | |
| RL5 | I know how to report a practice concern in the workplace | | | | | | |
| RL6 | I feel safe reporting professional behaviour that concerns me | | | | | | |
| RL7/JS | I feel comfortable approaching the Clinical Nurse Manager | | | | | | |
| RL8/JS | I feel comfortable approaching the Team Leader or equivalent | | | | | | |
| RL9/JS | I feel comfortable approaching my assigned Preceptor(s) | | | | | | |
| RL10/JS | I feel comfortable approaching the dedicated GNTP Coordinator | | | | | | |
| RL11/JS | I feel comfortable approaching the L&D Coordinator/Clinical Educator | | | | | | |
| RL12/JS | I feel comfortable approaching my Nursing/Midwifery coworkers | | | | | | |
| RL13/JS | I enjoy being a Nurse/Midwife | | | | | | |
| | **Relationships (RL) Emotional Assessment 10 items** | | | | | | |
| RL14/JS | I feel welcomed into the work place | | | | | | |
| RL15/JS | I take time to practice self-care | | | | | | |
| RL16/JS | My workplace recognises and supports my transition | | | | | | |
| RL17/JS/CO | I am able to manage the challenges of shift work effectively | | | | | | |
| RL18/JC/CO | I have been involved in a Clinical Incident | | | | | | |
| RL19/JS/CO | I worry about my responsibilities/ workload before beginning my shift | | | | | | |
| RL20/JS | I find it difficult to sleep between shift changes, as I am anxious about the next shift. | | | | | | |
| RL21/JC/CO | I have taken Personal Leave due to my issues/concerns | | | | | | |

| | | | | | | | | |
|---|---|---|---|---|---|---|---|---|
| | regarding my work | | | | | | | |
| RL22/ITS | I have considered leaving Nursing as a profession | | | | | | | |
| RL23/ITS | I plan to leave the profession of Nursing | | | | | | | |
| | **Knowledge (KN) 16 Items** | | | | | | | |
| KN1/CO | My formal education prepared me to practice as a professional Nurse/Midwife | | | | | | | |
| KN2/CO | I feel confident performing the clinical skills required of me | | | | | | | |
| KN3/CO | I feel confident performing clinical care to patients with complex needs | | | | | | | |
| KN4/CO/CJ | I feel confident responding to changes in the clinical status of my patients | | | | | | | |
| KN5/CO/CT | I feel confident in my ability to think critically | | | | | | | |
| KN 6/CO/DM | I feel confident escalating my concerns regarding the condition of my patients | | | | | | | |
| KN7/JS | My perceptions of the Nursing/Midwifery profession were accurate | | | | | | | |
| KN8/JS | I have the knowledge I need to practice Nursing/Midwifery | | | | | | | |
| KN9/JS | My workplace is invested in my ongoing learning and professional development | | | | | | | |
| KN10 | The Nurses/Midwives that I work with have realistic expectations of my clinical abilities | | | | | | | |
| KN11 | The Clinical Nurse Manager has a realistic expectation of my clinical abilities | | | | | | | |
| KN12 | The Team Leader has a realistic expectation of my clinical abilities | | | | | | | |

| | | | | | | | | |
|---|---|---|---|---|---|---|---|---|
| KN13 | I have a realistic expectation of my clinical abilities | | | | | | | |
| KN14/CJ | I am able to see how apparently unconnected activities are linked to make up an overall clinical picture | | | | | | | |
| KN/CO15 | I understand the three stages of Transition | | | | | | | |
| KN/CO16 | I know how to recognise Transition Shock | | | | | | | |

| | | |
|---|---|---|
| **CO – Confidence** | **DM Decision Making** | **CJ – Clinical Judgment** |
| **CT – Critical thinking** | **JS – Job Satisfaction** | **ITS – Intention to stay** |

These questions will be presented to the graduate nurses electronically through Survey Monkey three times throughout the 12 month program (both cohorts—National GNTP program and Comparison GNTP group).

Survey Intervals - 1 month, 5 months and 11 months.

Although the improvement in the quality of nursing staff is difficult to objectively quantify, I am hoping that by monitoring the graduate's **capability**, **confidence**, **responsibility** and **accountability** at set intervals throughout the program using this assessment tool, incremental improvements will be shown.

Data from these questions will determine if the hypothesis that graduates enrolled in the National Graduate Nurse Transition Program will emerge as responsible and accountable clinicians who, due to the supportive program based on the seminal transition research of Dr. J. Duchscher, will be able to practice in a confident and capable manner by the end of the program.
