# Supplementary material for: Growth and Adaptation of Newly Graduated Nurses Based on Duchscher’s Stages of Transition Theory and Transition Shock Model: A Longitudinal Quantitative Study
Source: Nurs Rep. 2025 Dec 9;15(12):437. doi: 10.3390/nursrep15120437 (PMC12736343; doi:10.3390/nursrep15120437)
Supplement: Supplementary file 1 [file nursrep-15-00437-s001.zip › SF Table S1 Descriptive.pdf]

[illegible]	[illegible]	[illegible]	[illegible]	[illegible]	[illegible]	[illegible]	[illegible]	[illegible]	[illegible]
56	3	6	5.02	0.618	30	4	6	5.30	0.596
56	2	6	4.59	1.172	30	2	6	4.83	0.986
56	2	6	4.59	0.987	30	2	5	4.03	1.033
56	1	6	2.43	1.399	30	1	6	2.47	1.502
54	4	6	4.98	0.658	29	4	6	5.14	0.639
54	4	6	5.63	0.560	28	4	6	5.50	0.638
54	4	6	5.50	0.607	29	4	6	5.48	0.634
54	3	6	5.13	0.778	29	4	6	5.28	0.751
54	2	6	3.63	0.996	29	2	6	4.31	1.105
54	3	6	4.87	0.702	29	4	6	5.17	0.602
36	2	6	4.92	0.770	17	4	6	5.06	0.556
54	4	6	4.91	0.524	28	3	6	5.00	0.667
54	3	6	4.56	0.744	29	4	6	4.72	0.649
54	3	6	4.74	0.705	29	3	6	4.86	0.639
54	3	6	4.74	0.757	29	3	6	5.14	0.833
54	3	6	4.54	0.665	29	4	6	4.93	0.530
54	3	6	5.00	0.644	29	4	6	5.14	0.581
54	3	6	4.65	0.705	29	2	6	4.90	0.900
54	3	6	4.76	0.751	29	3	6	5.00	0.756
54	2	6	4.11	1.341	29	2	6	3.83	1.391
54	1	6	3.33	1.505	29	1	5	2.93	1.361
54	2	6	3.56	0.883	29	2	6	4.14	1.060
54	3	6	4.98	0.629	29	4	6	5.17	0.539
34	3	6	4.94	0.547	18	4	6	5.22	0.647
54	2	6	4.52	0.795	28	4	6	4.79	0.630
54	2	6	5.04	0.800	28	3	6	5.00	0.770
54	2	6	4.44	0.904	29	2	6	4.93	0.884
54	2	6	5.04	0.889	29	5	6	5.48	0.509
54	4	6	5.35	0.588	29	4	6	5.45	0.632
49	4	6	5.33	0.658	25	4	6	5.52	0.653
51	2	6	5.31	1.140	28	3	6	5.43	0.790
54	2	6	5.09	1.069	29	3	6	5.03	1.017
54	4	6	5.30	0.603	29	4	6	5.55	0.572
54	3	6	5.24	0.725	29	3	6	5.28	0.841
54	3	6	5.15	0.684	29	4	6	5.31	0.541
54	3	6	4.89	0.839	28	3	6	4.93	0.766
54	3	6	5.00	0.801	29	2	6	5.17	0.928
54	3	6	4.80	0.595	29	3	6	5.03	0.680
54	1	6	4.13	1.388	28	1	6	3.71	1.560
54	1	6	4.07	1.330	29	2	6	3.55	1.121
54	1	6	3.48	1.514	29	2	6	3.07	0.998
54	1	6	1.94	1.106	29	1	6	2.21	1.473
54	1	6	2.11	1.192	29	1	4	1.90	0.900
53	1	3	1.49	0.639	29	1	3	1.59	0.628
54	2	6	4.28	0.899	29	2	6	4.14	1.156
54	3	6	4.80	0.737	29	3	6	4.97	0.731
54	3	6	4.50	0.746	29	3	6	4.69	0.850
54	3	6	4.63	0.653	29	4	6	4.90	0.618
