# Supplementary material for: Growth and Adaptation of Newly Graduated Nurses Based on Duchscher’s Stages of Transition Theory and Transition Shock Model: A Longitudinal Quantitative Study
Source: Nurs Rep. 2025 Dec 9;15(12):437. doi: 10.3390/nursrep15120437 (PMC12736343; doi:10.3390/nursrep15120437)
Supplement: Supplementary file 1 [file nursrep-15-00437-s001.zip › SF Table S2 PCA Factor Descriptions.pdf]

s Three Stages

	Phase 1 (0–4.5 months) Factor & Description	Phase 2 (5–7 mor
	F1: .590 → Confidence & Role Clarity	F4: .692 → Role U
	F5: .811 → Stress & Misalignment	F2: –.413 / F4: –.5
	F2: .735 → Confidence in Team Interaction	F3: .809 → Comm
	F2: .744 → Team Communication Confidence	F2: .417 / F3: .647<br>Communication
	F2: .862 → Multidisciplinary Teamwork	F3: .608 → Collab
	F1: .461 / F2: .495 → Mixed: Confidence/Team Role	F1: .697 → Clinica
	F3: –.742 → Role Confusion	F1: –.595 → Confi
	F1: .431 / F2: .611 → Mixed: Confidence & Clinical Judgment	F1: .832 → Indepe
	F2: .554 / F5: .446 → Team Confidence & Emotional Labor	F1: .523 → Confid
	F1: .570 / F2: .413 → Adaptability & Self-Efficacy	F1: .753 → Profes
	F1: .585 / F5: –.474 → Resilience / Inverse Stress	F2: .449 → Compo
	F1: .739 → Resilience	F2: .808 → Persev
	F1: .812 → Adaptability in Practice	F2: .710 / F1: .474
	F4: .691 → Workplace Engagement	F3: .513 → Social
	F5: .810 → Work-Life Spillover & Stress	F4: –.748 → Job D
	F5: .815 → Turnover Intention & Stress	F4: –.488 / F2: –.4
m Leader, Coworkers, Assistant in Nursing)	F1: .482 → Role Differentiation & Understanding	F1: .782 → Role U
ursing (AIN)	F2: .647 → Role Clarity & Differentiation	F2: .553 → Role U
coworkers (e.g., Clinical Nurse Manager, Clinical Nurse,	F2: .673 → Role Differentiation	F2: .658 → Role D
/Midwife and an Enrolled Nurse	F3: .407 → Accountability & Responsibility	F3: .464 → Accour<br>F4: .561 → Accour
	F1: .763 → Leadership	F1: .595 → Leade<br>F5: –.465 (Negativ
	F1: .777 → Respect & Role Clarity	F2: .657 → Respe
	F1: .720 → Respect & Workplace Interaction	F2: .572 → Respe
	F1: .852 → Respect	F3: .516 → Respe
	F1: .573 → Respect & Team Interaction	F2: .575 → Respe
	F2: –.488 → Role Misalignment	F1: .745 → Role A
	F1: .698 → Professional Confidence	F1: .601 → Workp
y situation	F2: .680 → Problem-Solving	F3: .458 → Analyti
	F1: .665 → Workplace Wellbeing	F1: .597 → Workp
	F2: .455 → Work-Life Balance	F2: .597 → Work-L
any personal blame	F1: .796 → Feedback Reception	F1: .571 → Feedba
	F3: –.700 → Emotional Struggles	F2: .606 → Role A
/Midwife	F2: .781 → Emotional Struggles & Role Doubts	F2: .607 → Role U
	F1: .863 → Leadership & Workplace Acceptance	F1: .417 → Leade
