# Supplementary material for: Growth and Adaptation of Newly Graduated Nurses Based on Duchscher’s Stages of Transition Theory and Transition Shock Model: A Longitudinal Quantitative Study
Source: Nurs Rep. 2025 Dec 9;15(12):437. doi: 10.3390/nursrep15120437 (PMC12736343; doi:10.3390/nursrep15120437)
Supplement: Supplementary file 1 [file nursrep-15-00437-s001.zip › SF Table S3 Chi Square.pdf]

	3.573	4	0.467
	8.529	4	0.074
	14.996	4	0.005
ng Nurse/Midwife	24.477	4	<.001
	7.551	4	0.109
	9.532	4	0.049
	12.401	4	0.015
	16.205	4	0.003
	5.392	4	0.249
d	5.843	4	0.211
	4.289	4	0.368
	5.458	4	0.243
	0.632	4	0.959
Coworkers, Assistant in Nursing)	2.813	2	0.245
l)	0.547	2	0.761
(e.g., Clinical Nurse Manager, Clinical	5.78	4	0.216
d an Enrolled Nurse	6.896	4	0.142
	17.731	4	0.001
	13.307	4	0.01
	3.043	4	0.551
	2.849	4	0.583
	4.054	4	0.399
	6.337	4	0.175
	5.872	4	0.209
	17.435	4	0.002
	5.54	4	0.236
	7.909	4	0.095
nal blame	5.144	4	0.273
	6.235	4	0.182
	2.37	4	0.668
	26.462	4	<.001
	5.887	4	0.208
	0.946	4	0.918
	3.542	4	0.472
	2.622	4	0.623
	6.55	4	0.162
	9.883	4	0.042
	2.82	4	0.588
	2.489	4	0.647
	4.056	4	0.398
	3.048	4	0.55
	1.744	2	0.418
	0.751	4	0.945
	4.776	4	0.311
	[illegible]	[illegible]	[illegible]

	[illegible]	4	[illegible]
	8.998	4	0.061
	11.303	4	0.023
	8.454	4	0.076
	4.151	4	0.386
	6.781	4	0.148
	2.761	4	0.599
es	5.412	4	0.248
	6.283	4	0.179
	2.883	4	0.578
	8.62	4	0.071
erall clinical picture	2.46	4	0.652
	2.842	4	0.585
	4.359	4	0.36
