# Supplementary material for: Growth and Adaptation of Newly Graduated Nurses Based on Duchscher’s Stages of Transition Theory and Transition Shock Model: A Longitudinal Quantitative Study
Source: Nurs Rep. 2025 Dec 9;15(12):437. doi: 10.3390/nursrep15120437 (PMC12736343; doi:10.3390/nursrep15120437)
Supplement: Supplementary file 1 [file nursrep-15-00437-s001.zip › SF Table S4 Logistics Regression.pdf]

: Predictors of Graduate Nurse and Midwife Transition Stage

	Chi-Square	df	p-value
g Nurse/Midwife	3.959	2	0.138
ctations	3.416	4	0.491
	1.38	4	0.848
	2.872	4	0.58
	2.102	4	0.717
e	5.142	4	0.273
	18.112	4	0.001
	4.28	4	0.369
	5.241	4	0.263
	8.084	4	0.089
	11.177	4	0.025
	0.796	4	0.939
	1.883	4	0.757
	8.505	4	0.075
	3.298	4	0.509
	2.719	4	0.606
linical Nurse Manager, Team Leader, Coworkers, Assistant in Nursing)	2.822	2	0.244
nd that of an Assistant in Nursing (AIN)	1.235	2	0.539
nd that of my senior nurse coworkers (e.g., Clinical Nurse Manager,	4.994	4	0.288
etween a Registered Nurse/Midwife and an Enrolled Nurse	3.09	4	0.543
	1.759	4	0.78
work with	6.297	4	0.178
I work with	8.874	4	0.064
vork with	8.319	4	0.081
vork with	4.053	4	0.399
would be like	4.358	4	0.36
ng intimidated	0.68	4	0.954
mation the core issue in any situation	27.538	4	0
work in perspective	2.602	4	0.626
vork life	10.705	4	0.03
n coworkers without feeling any personal blame	10.988	4	0.027
re difficult than I expected	4.361	4	0.359
ecision to become a Nurse/Midwife	9.545	4	0.049
e	25.59	4	<.001
vork with	5.408	4	0.248
at I work with	1.57	4	0.814
ork with	7.154	4	0.128
workplace	0	4	1
t concerns me	6.745	4	0.15
se Manager	0	4	1
er or equivalent	0	2	1
receptor(s)	0	2	1
GNTP Coordinator	0	4	1
nator/Clinical Educator	6.824	4	0.146
lwifery coworkers	26.223	2	<.001

	0	2	1
	0	2	1
	4.159	4	0.385
nsition	12.76	4	0.013
rk effectively	7.427	4	0.115
	36.64	4	<.001
re beginning my shift	10.494	4	0.033
as I am anxious about the next shift	25.483	4	<.001
/concerns regarding my work	4.162	4	0.384
sion	0	2	1
	0	0	1
s a professional Nurse/Midwife	10.19	4	0.037
quired of me	2.123	4	0.713
nts with complex needs	1.621	4	0.805
inical status of my patients	4.272	4	0.37
	2.239	4	0.692
ng the condition of my patients	5.009	4	0.286
ssion were accurate	7.46	4	0.113
g/Midwifery	4.288	4	0.368
ng and professional development	7.356	4	0.118
alistic expectations of my clinical abilities	1.108	4	0.893
pectation of my clinical abilities	1.107	4	0.893
f my clinical abilities	4.137	4	0.388
ities	10.858	4	0.028
activities are linked to make up an overall clinical picture	7.274	4	0.122
	0.818	4	0.936
	7.015	4	0.135
